# Comparison of Major Compounds in Essential Oils Steam Distilled from Fresh Plant Material of South African Hop Varieties

**DOI:** 10.3390/life15020282

**Published:** 2025-02-12

**Authors:** Olga de Smidt, Wanda du Plessis, Puleng Rose Zacharia, Idah Tichaidza Manduna

**Affiliations:** 1 Centre for Applied Food Sustainability and -Biotechnology (CAFSaB), Central University of Technology (CUT), Bloemfontein 9300, South Africa; pzacharia@cut.ac.za (P.R.Z.); imanduna@cut.ac.za (I.T.M.); 2 Preserve Botanica PTY, Ltd., George 6529, South Africa; wanda@preservebotanica.co.za

**Keywords:** hops, hop essential oils, terpenes, GC-FID, GC-MS, aliphatic compounds, toxicity

## Abstract

Apart from the importance of bittering acids in the brewing sector, hops also produce terpene-rich essential oils, recognised for their therapeutic benefits. Agri-processing practices of this crop in South Africa produce tonnes of discarded (waste) plant material that could still contain sufficient bioactive compounds to justify upcycling. This research aimed to determine the chemical composition of steam distilled essential oils from fresh hop plant material destined for disposal. Essential oils from eight hop varieties unique to South Africa were produced on industrial scale using steam distillation. Chemical profiling was performed using GC-FID and MS. A total of 208 compounds were identified and oil consisted largely of terpenes (89.04 ± 1.89%) as well as aliphatic esters and -ketones (6.1 ± 1.15%). Myrcene (27.8–48.15%) was the most abundant monoterpene and α-humulene (19.52–24.98%), β-caryophyllene (8.47–13.73%) and β-farnesene (2.08–7.57%) constituted the majority of the sesquiterpenes fraction. Experimental variety XJA2/436 had the highest myrcene fraction (48.15%) and its chemical composition was markedly different from the other varieties. The major compounds in African Queen hop oil were methyl (4Z) decanoate (0.74%), 2-tridecanone (0.77%) and β-farnesene (7.57%). Southern Dawn hop oil contained the highest fractions of 2-undecanone (1.21%) and α-humulene (24.89%) and Southern Passion hop oil contained the highest β-caryophyllene fraction (13.73%). These findings established that fresh hop vegetative biomass shows promise to be transformed into a valuable resource.

## 1. Introduction

Current global trends in health awareness and environmental stewardship are driving an increase in the demand for natural products, including those derived from medicinal and aromatic plants (MAPs). MAPs are preferred because they are considered natural, relatively safe and environmentally friendly [1], as well as for their role in traditional or folk medicine [2]. The therapeutic, cosmetic, nutraceutical and other benefits derived from plants are due to thousands of secondary plant metabolites that are produced from metabolic pathways that breakdown carbohydrates, lipids, nucleic acids and proteins (the primary metabolites). While primary metabolites ensure fundamental processes for plant survival such as growth, development and reproduction, secondary metabolites facilitate long-term survival by providing protection against disease and predation, attracting pollinators and allelopathy [3].

Essential oils are an important group of secondary metabolites whose diverse chemistry and biological properties have become more relevant and have sparked renewed research interest [4,5]. They are complex mixtures of volatile, lipophilic, and scented compounds, mainly terpenes, phenols, aliphatic alcohols, aliphatic ketones and aliphatic esters [2,6,7]. The most abundant group, terpenes, are biosynthesised through the mevalonate pathway while the phenolic compounds are produced from the shikimate pathway [4]. Essential oils can be extracted from plant material by steam distillation, expression, hydrodistillation, hydrodiffusion, subcritical water, supercritical CO_2_, solvent free microwave, solvent extraction and solvent-steam extraction and enfleurage [2,8]. The phytochemical profile and biological activities of essential oils differ according to the plant species, genetic composition of the plant, the plant part, method of extraction as well as climate and growth conditions of the plants [9].

Only about 300, i.e., 10% of known essential oils are of commercial importance with use as medicines, in agriculture, cosmetics and food [7]. Therefore, research has barely scratched the surface of these resources and further evaluation of other essential oils from other plants and their chemical constituents is warranted to allow for better and specifically directed applications and new discoveries. An example of plants that have generated a lot of interest in the essential oil industry is hemp, *Cannabis sativa* L. (Cannabaceae). Ever since the cultivation of hemp was authorised in different regions of the world, 2001 in Europe [10] and 2017 in South Africa [11], strides have been taken to exploit the plant and its essential oils for food, medicine and fibre among other applications. However, cannabis use and cultivation remain controversial in African countries, including South Africa. Growth potential for the cannabis industry is limited by the legal requirement for licenses for cultivation, for research as well as for medicinal use. Additionally, the presence of the phytocannabinoids [10], cannabidiol (CBD) and tetrahydrocannabinol (THC) requires control and guidelines by the South African Health Products Regulatory Authority [12] that are prohibitive to many stakeholders. Consequently, attention can be turned to hops *Humulus lupulus* L., a plant that also belongs to the Cannabaceae family and is closely related to *C. sativa*. In fact, Ovidi et al. [13] compared the essential oils from Italian cultivars of hemp and hops using Headspace–Gas Chromatography–Mass Spectrometry (HS-GC-MS) and found that the oils of both plants were mainly composed of the terpene derivatives namely β-myrcene, limonene, β-caryophyllene and α-humulene.

Hops are mainly used in the brewing industry where the addition of hop cones gives beer its distinctive flavour and aroma while also preserving its microbiological stability [14,15]. Prior to the advent of beer brewing, archaeological findings place hop seeds in the Neolithic period when hops were probably only used for medicinal purposes. There are records of similar historical use of hops across cultures (Arab, Ayuverdic Indian, Native American) for various conditions which attest to the medicinal efficacy of the plant [16,17]. Hops were used for their anti-inflammatory, soothing, digestive and antimicrobial properties. Additionally, hops were used against fevers, for spleen disorders, ear infections, toothaches, sleep disorders, stomach complaints, tuberculosis, coughs, colds, wounds, and leprosy. Some of these therapeutic properties, especially the soothing/calming effect of hops and their use as a sleeping agent were attributed to the volatile components of hop oils [16,17,18].

Commercial hop production in South Africa started in the 1970s mainly to serve the beer industry. In the South African hop farming sector, the vegetative biomass (1.0–1.5 tonnes estimated per hectare) that remains after use of hop cones is typically regarded as waste and used for composting. This plant material that remains after picking of the hop cones holds immense potential as a matrix for the extraction of bioactive compounds [15] that have a multitude of applications in the pharmaceutical, cosmetic and food industries. Accordingly, this has potential to boost the South African Bioeconomy in a similar fashion as that predicted for the USA, where the pharmacological use of bioactive compounds found in hops could potentially increase the role of the hop industry in the performance of the US economy. Using salvaging processes to recover hop oils from ‘discarded’ plant material could contribute to the environmental sustainability of hop farming. For this to happen, South African hop oils need to be evaluated for their phytoconstituents, which will allow for the determination of potential applications. The aim of this study was to profile and compare the chemical composition of essential oils steam distilled from fresh hop plant material (leaves, cones and bract) considered only a co-product of the brewing industry that was destined for disposal.

## 2. Materials and Methods

### 2.1. Sampling Area and Hop Varieties

South African hop varieties are grown in the Waboomskraal Valley, a farming community north of George (33.9778° S, 22.4953° E) in the southern region of the Western Cape Province. The area has shallow to moderately deep soil derived from Table Mountain sandstone. The local vegetation is characterised by fynbos communities of Proteaceae, Ericaceae Restionaceae and Cyperaceae in the valleys and Afromontane Forest on higher ground. Annual precipitation averages 800 mm in the valley and 1100 mm in the mountains [19]. Hop production from 11 farms (7 are independently owned) goes solely to South African Breweries (SAB) for processing and application in the beer sector. The varieties studied were African Queen, experimental variety XJA2/436, Southern Sublime, Southern Aroma, Southern Dawn, Southern Passion, Southern Promise, and Southern Star.

### 2.2. Oil Distillation and Processing

The hop essential oils used for this experiment were manufactured during two hop harvest seasons in March (2023 and 2024) by salvaging fresh hop biomass from three farms. Essential oils were steam distilled on industrial scale from fresh leaves, cones and fine bract. A total of 28.6 and 12.7 tonnes of hop biomass were processed during 2023 and 2024, respectively. The Preserve Botanica standard operating and manufacturing procedures were followed [20]. A 540 L stainless steel vessel was layered with freshly harvested plant biomass. Wet steam at a temperature not exceeding 85 °C at an average run time of 77.24 ± 15.83 min transported hydrophobic oils on the surface of water droplets up in the vessel. Oils were then separated from the water-based hydrosol during the condensing phase. A total of 385 and 154 cycles were completed from the beginning to the end of 2023 and 2024 harvest seasons, respectively. Variety specific steam distilled hop oils were lotted and batched and randomly selected batches were analysed. A ratio of 60:30 bract to leaf and cone were processed to produce an average yield of 0.8 g oil/100 g hop biomass.

Refinement of oils entailed filtering the crude oils through a 75 µm stainless steel mesh and separation from the remaining hydrosol in a glass separation funnel for 1 h, followed by 24 h of cooling at 4 °C [21]. Final separation was performed by centrifugation at 1500× *g* for 10 min followed by decanting into 1 L aluminium bottles. All oil samples were stored at 4 °C.

### 2.3. Physicochemical Measurements and Gas Chromatography Analyses

Refractive indices were measured on an Abbemat 350 refractometer (Anton Paar, Graz, Austria) at 20 °C. Relative density was determined at 20 °C with a 10 mL pycnometer equipped with a thermometer against a reference of fresh demineralised water. Optical rotation was measured on neat oils on an Autopol IV, model A21200 APIV/1W, digital polarimeter (Rudolph Research Analytical, Hackettstown, NJ, USA) using a 100 mm cell.

Quantitative analysis of the chemical composition was performed by Laboratoire PhytoChemia (Saguenay, QC, Canada) using FAST Gas chromatography-flame ionisation detector (GC-FID) (ISO/IEC 17025:2017 [22] accredited method PC-MAT-014). GC-FID analyses were carried out on an Agilent 7890A GC (Agilent, Technologies, Santa Clara, CA, USA) featuring a split/splitless injector and two FID detectors. Columns: DB-5 (10 m × 0.10 mm × 0.10 μm, Agilent Technologies, Santa Clara, CA, USA); DB-Wax (10 m × 0.10 mm × 0.10 μm film thickness, Agilent Technologies). Temperature program: 35 °C for 1 min, then, 9 °C/min up to 250 °C. Injection port: 250 °C. Injection volume: 0.03 μL for essential oil. Initial inlet pressure: 216.5 kPa, constant flow mode. Carrier gas: H_2_, flow rate: 0.7 mL/min and injection mode: split (300:1). FID (250 °C): H_2_ flow: 40 mL/min; air flow: 450 mL/min; makeup flow (N_2_): 45 mL/min. Sampling rate: 0.01 min. Injections were also performed in parallel on an Agilent 7890B GC featuring a split/splitless injector coupled to an Agilent 5977B mass spectrometer. Column: HP-5MS 30 m × 0.25 mm × 0.25 μm film thickness (Agilent Technologies), or DBWaxUI 30 m × 0.25 mm × 0.25 μm film thickness. Temperature program: 40 °C for 2 min, 3 °C/min up to 220 °C, followed by, 220 °C for 2 min. Injection port: 250 °C. Injection volume: 0.5 μL. Initial inlet pressure: 48.7 kPa (constant flow mode). Carrier gas: He, flow rate: 1.0 mL/min. Injection mode: split (200:1). MS interface temp.: 250 °C; MS mode: EI; mass range: 40–550 u; scan speed: 1562 u/s [23]. Trace concentrations of compounds detected below 0.005% of the total signal were not included in the data set. Where two or more compound percentages could not be solved due to coelution, the compounds were not included in the dataset. These compounds included: 2-methylbutyl propionate, isoamyl propionate, (Z)-tridecenone isomer I, 2-nonanone, methanethiol hexanoate, geranyl propionate, selina-4(15),7(11)-diene, trans-cadina-1,4-diene, α-amorphene and γ-muurolene.

### 2.4. Data Processing

Data were processed by Laboratoire PhytoChemia using Unichrom (FID data) and MassHunter Qualitative Analysis B.07.00 (MS data). A semi-automatic process applying an algorithm was used to collapse data acquired from the multi-columns system into a single set of consolidated contents. In case of discrepancies between columns, the algorithm is set to prioritise data from the most standard DB-5 column, and smallest values as to avoid overestimating individual content. Compounds were identified from their retention indexes as calculated from C7 to C40 alkane standards and/or from MS databases NIST17 & Adams et al. [24] and custom libraries built from pure compounds and commercial essential oils) and through systematic comparison of MS and FID chromatograms for both apolar and polar columns. For essential oils, semiquantitative data were obtained from the FID detector response on the DB-5 column without any correction factor, using the internal normalisation method [23]. Principal Component Analysis (PCA) was conducted using the Scikit-learn library in Python based on chemical class data namely aliphatic compounds (esters, ketones, alcohols, aldehydes, alkanes), Terpenes (di-, mono-, sesqui-) as well as their esters, ethers, alcohols, aldehydes.

## 3. Results

The feed material used in this study for steam distillation of essential oils was considered a byproduct of agri-processing and would either have been composted or sent to a landfill. The average oil yield was 0.8 g oil/100 g hop biomass. Bract and leaf volumes and oil yields varied from beginning to end of harvest season, for different farms, varieties, and seasons.

Analysis of the produced volatile oils confirmed that they were not adulterated (no contaminant or diluent detected). Refractive indices were high, 1.483 ± 0.0024, and within the expected range of 1.43–1.61 for volatile oils (Table 1) [25]. GC-FID analysis of oils from the different hop varieties detected a total of 208 known compounds which represented 94.9–98.3% of the total oil and several unknown (unidentified) compounds. Unknown fractions ranged from 0.95 to 1.6%. Experimental variety XJA2/436 had the lowest number of unknown compounds and Southern Sublime the highest. Results were firstly organised in functional groups, as depicted in the Table 1, to assess similarity. The most abundant groups were terpenes, esters and ketones. Experimental variety XJA2/436 produced oil with the highest fraction of terpenes (89.81%), Southern Aroma and Southern Dawn oils had the highest fraction of ketones (3.52%) and oil from Southern Sublime contained the highest fraction of esters (5.64%). The functional groups did not highlight marked differences in varieties and a more detailed analysis of the chemical compound classes was necessary.

**Figure 1:**
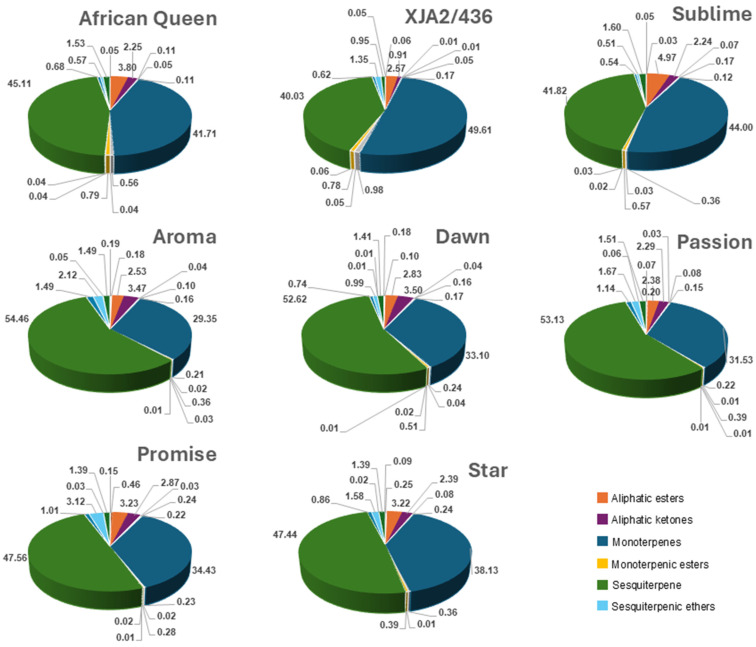
Chemical composition of each South African hop variety for aliphatic compounds (esters, ketones, alcohols, aldehydes, alkanes), Terpenes (di-, mono-, sesqui-) as well as their esters, ethers, alcohols, aldehydes). Compounds with fractions >1% in at least one hop variety were colour-coded in the figure legend for easy reference.

Comparison of the different classes included aliphatic ester, -ketones, -aldehydes, -alcohols, alkanes, di-, mono-, sesquiterpenes and their alcohols, aldehydes, esters ethers and oxygenated forms. Figure 1 illustrates mono- and sesquiterpenes to be present in the highest fractions with notable differences in distribution amongst varieties. Oil from experimental variety XJA2/436 had the highest fraction of monoterpenes (49.61%) and Southern Aroma the highest fraction of sesquiterpenes (54.46%). Southern Sublime oil had higher fractions of sesquiterpenic esters (4.97%) and XJA2/436 notably lower aliphatic ketones (0.91%). Sesquiterpenic ether fractions were slightly higher in Southern Promise (3.12%) compared to the other varieties. The full dataset can be viewed in Supplementary Table S1.

**Figure 2:**
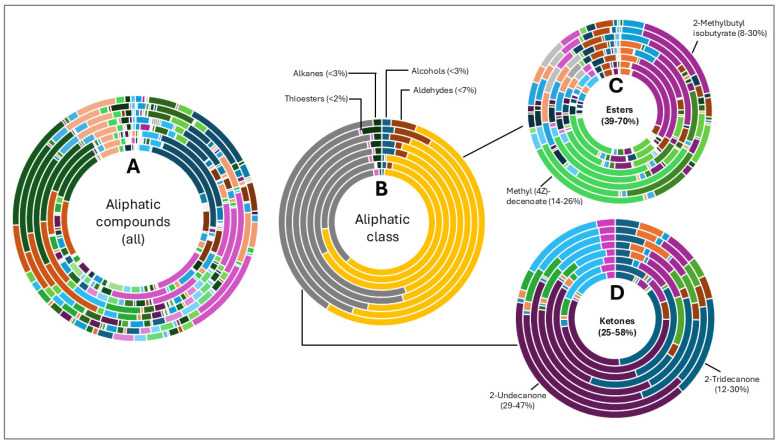
Aliphatic compound distribution in 8 hop oils. Inner to outer rings represent African Queen, XJA2/436, Southern Sublime, Southern Aroma, Southern Dawn, Southern Passion, Southern Promise and Southern Star. (**A**) represents all the aliphatic compounds and serves to demonstrate their diversity in the hop oils. (**B**) Aliphatic compounds grouped according to class. The percentages indicated were calculated fractions of the total aliphatic compounds only. (**C**) represents the ester and (**D**) ketone components of the oils. 2-Methylbutyl isobutyrate and methyl (4Z)-decenoate were the two most abundant esters and 2-undecanone and 2-tridecanone the two most abundant ketones recorded.

The hop oils had a diverse distribution of aliphatic compounds as can be seen in Figure 2A. When grouped according to class, compounds in the highest fractions were esters (39–70%) and ketones (25–58%) evident in Figure 2C and Figure 2D, respectively. Fractions of other aliphatic classes were less than 7%. The two most abundant esters found in hop oils for all 8 varieties were 2-methylbutyl isobutyrate and methyl (4Z)-decenoate. Dominant ketones were 2-undecanone and 2-tridecanone, andoil from experimental variety XJA2/436 did not contain 2-tridecanone.

**Figure 3:**
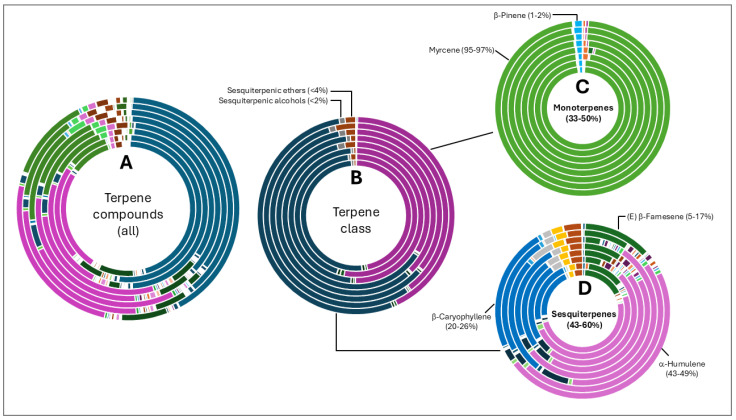
Terpene compound distribution in 8 hop oils. Inner to outer rings represent African Queen, XJA2/436, Southern Sublime, Southern Aroma, Southern Dawn, Southern Passion, Southern Promise and Southern Star. (**A**) represents all the terpene compounds and serves to demonstrate their diversity in the hop oils. (**B**) Terpene compounds grouped according to class. Percentages indicated in (**C**,**D**) were calculated fractions of the total terpene compounds only. (**C**) represents the monoterpenes and (**D**) sesquiterpene components of the oils. Myrcene is the most abundant monoterpene present. *α*-humulene, *β*-caryophyllene, and (E) β-farnesene were the sesquiterpenes present in the highest fractions in all 8 oil extracts.

Terpene fractions were not less diverse than the aliphatic compounds, although the dominance of specific classes were more evident. Total terpene compound diversity is shown in Figure 3A and grouped according to class in Figure 3B. Monoterpenes (35–50%) and sesquiterpenes (43–60%) were the most abundant compounds with other classes such as ethers and alcohols forming <4% of the fraction. Monoterpenes were almost solely represented by myrcene (95–97%). Three sesquiterpene compounds were abundant in oils from all varieties namely α-humulene, β-caryophyllene and (E) β-farnesene.

**Figure 4:**
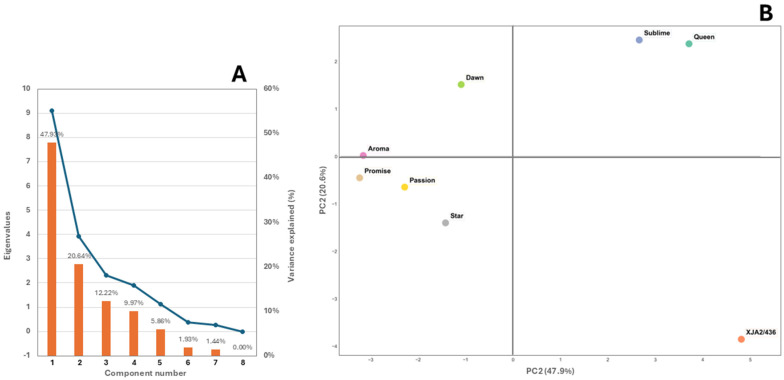
(**A**) Scree plot depicting the eigenvalues of the principal components, where the x-axis represents the component number. The blue line on the y-axis shows the corresponding eigenvalue and bars indicate the % variance explained. (**B**) Principal components analysis (PCA) performed on the major chemical classes for the eight South African hop varieties African Queen, XJA2/436, Southern Sublime, Southern Aroma, Southern Dawn, Southern Passion, Southern Promise and Southern Star.

The scree plot in Figure 4A indicates that 96.62% of the variation among varieties could be attributed to aliphatic alcohols, aldehydes, esters, ketones and sulphides. Chemical structure of the populations by PCA based on the 19 detected chemical classes showed that the first two PCA axes represented 68.57% of the total variance (Figure 4B). Three distinct groups were noticeable with experimental variety XJA2/436 grouping separate from the other varieties and African Queen and Southern Sublime also distant from the remaining 5 varieties.

Aliphatic terpenes were the most abundant compounds in oils from all eight hop varieties. Total fractions of aliphatic compounds were 6.29, 3.66, 7.53, 6.51, 6.80, 5.06, 6.98 and 6.03% and terpenes 89.65, 93.65, 88, 88.27, 8.44, 88.31, 86.93, and 89.04% for African Queen, XJA2/436, S. Sublime, S. Aroma, S. Dawn, S. Passion, S. Promise and S. Star, respectively. Table 2 presents characteristics, including toxicity, of selected compounds from the aliphatic and terpene class that were abundant in all 8 varieties as identified in Figure 2 and Figure 3. Summary data for each compound; group, CAS numbers, formula, molecular weight and structures were obtained from the PubChem database (https://pubchem.ncbi.nlm.nih.gov (accessed on 15 November 2024) [26].

## 4. Discussion

### 4.1. Chemical Profiling

Essential oil quality, quantity, and composition are subject to environmental conditions such as climate, cultivation, plant organ and soil composition. Despite consisting of a narrower range of volatile molecules compared to extracts, essential oils can still contain more than 300 different substances [6,7]. The chemical profiles of the essential oils distilled from 8 varieties of South African hop biomass contained 208 known compounds. Although the oils were steam distilled from discarded vegetative biomass the average yield was still 0.8 g oil/100 g hop biomass, which is comparable to the oil yield stipulated on the product information available for commercial hop pellets or these same varieties.

Hop oils were extracted from fresh hop leaves, cone and bract and comparisons of the chemical profiles of hop oils from fresh plant material and other hop oils from dried plant material were difficult since distillation in other studies were often performed on lab scale and from dried cones or pelletised hops [39,40,41]. However, the primary volatile constituents were similar to other studies, namely monoterpene β-myrcene, and the sesquiterpenes β-caryophyllene and α-humulene [42,43]. The distribution of different fractions could be subject to many factors mostly related to environmental conditions, source material and extraction method employed [44,45,46,47]. β-myrcene fractions were notable higher in the oil from South African varieties (27.8–48.2%) and humulene was the most abundant sesquiterpene detected in the oils [48]. Farnesene fractions in hop oils distilled from discarded vegetative biomass were higher compared to oils from international varieties (S.S. Steiner interpretation), but proper comparative analyses would have to be performed to verify this finding. Hop leaves have recently been considered as a source of bioactive compounds, but this study referred to products from solvent extraction and not steam distillation [15].

Aliphatic compounds (alcohols, aldehydes, esters, ketones and sulphides) accounted for 96.62% of the variation in oil chemical composition amongst the varieties. This diversity is also evident in the distribution of individual compounds in Figure 2A. The separate grouping of the experimental variety XJA2/436 was unexpected since it shares the same parentage as Southern Star (SAB product information unpublished).

### 4.2. Bioactivity and Toxicity

Extracts and oils from *Humulus lupulus* (hop) have been subjected to systematic research over the last 20 years. The longest topic studied is most likely the sedative effect of hops. Compounds mainly responsible for sedative effects are bitter resins and essential oils. Most investigated properties of essential oils are antioxidant, anti-inflammatory, antimicrobial, wound-healing and anxiolytic activities [49]. The remainder of the discussion section focusses on the bioactivity and toxicity (Table 2), if any, associated with the most abundant aliphatic esters and ketones as well as terpenes present in South African hop oils.

#### 4.2.1. Aliphatic Esters and -Ketones

While there were 67 aliphatic compounds (17 ketones and 50 esters) detected in the hop oils used in this study, they only comprised 6% of total oil fractions. 2-Undecanone or methyl nonyl ketone is typically the most abundant aliphatic ketones in hop oils [50,51,52] and other plants such as ginger and herbs from the *Ruta* genus. This compound is used as a flavour agent in food and cosmetics. Studies have indicated insecticidal, acaricidal [53], antibacterial, antifungal, antiviral (against HSV-1, and HIV-1), anthelmintic and cytotoxic activity [54]. Studies on BALB/c mice and H9C2 cells have shown that 2-undecanone could also reduce inflammatory damage of mouse myocardium [55]. Additionally, Wu et al. [56] demonstrated that 2-undecanone may have nephroprotective activity through an anti-inflammatory effect produced by initiating mitophagy which suppresses Akt1-mTOR signalling in HEK 293 cells.

#### 4.2.2. Terpenes

Terpenes are a diverse family of natural products synthesised by plants, with approximately 55,000 members [7,57]. Most terpenes are found in the essential oil fraction of plant oils, and they can be described as aromatic hydrocarbon molecules produced by almost every plant, and some animals, for interaction with other organisms [58]. A total of 58 different terpenes, 16 mono- and 42 sesquiterpenes, were present in South African hop oils, the most abundant compounds were the monoterpene myrcene (27.8–48.15%) and sesquiterpenes *α*-humulene (19.52–24.98%), *β*-caryophyllene (8.47–13.73%) and *β*-farnesene (2.08–7.57%).

Hop essential oils have therapeutic effects due to the presence of terpenes and terpenoids in their composition [59]. At present, several health benefits have been attributed to the essential oil component of hops. Although most studies report on the health effects of the individual compounds, not the essential oil itself, the positive effects of hop oil constituents are not limited to only their described properties. Rutkin et al. [50] summarised the health benefits of selected hop constituents, including myrcene, *α*-humulene, β-caryophyllene as having sedative-, anti-cancer, analgesic-, anti-inflammatory and antioxidant properties.

##### Monoterpenes

Myrcene is an acyclic terpene hydrocarbon with a green, herbaceous, resinous and fresh hop aroma [42]. Myrcene has anti-catabolic and anti-inflammatory properties through inhibition of fats surrounding internal organs. Nuutinen [60] demonstrated the neuroprotection properties by preventing the oxidation of lipids and fatty acids that resulted in the creation of thiobarbituric acid reactive substances (TBARS), which shield the heart, brain, and other tissues from oxidative damage. Antimicrobial capabilities of β-myrcene were comprehensively reviewed by Duarte et al. [42] and showed activity against Gram-positive and Gram-negative bacteria, as well as selected fungal species. Despite its many therapeutic uses, *β*-myrcene is said to be a possible carcinogen and its food additive status was removed in 2018. This removal was hotly contested by Felter et al. [61]. Researchers called for a new interpretation of the language of the Delaney Clause, and to incorporate greater emphasis on the application of scientific understanding to refrain from classifying non-genotoxic chemicals as carcinogens.

##### Sesquiterpenes

The hydrocarbons α-humulene and β-caryophyllene are among the most important compounds in hop essential oil due to their antioxidant properties. These compounds have been tested for their ability to eliminate radicals and prevent lipid oxidation, with caryophyllene demonstrating stronger antioxidant properties than humulene [17]. Humulene is also called α-caryophyllene, but it does not contain the cyclobutane ring, and has a spicy or woody aroma [42]. A systematic review by Mendes de Lacerda Leite et al. [35] on the pharmacological and toxicological activities of *α*-humulene reported that 41% of the investigated articles described α-humulene and its isomers to have antitumour activity, followed by anti-inflammatory and antimicrobial activities (20% for both), other pharmacological activities (15%) and potential toxic (2%) activities. Mendes de Lacerda Leite et al. [35] mentioned solely one publication from the 1980s on the possible toxicology of *α*-humulene [34]. This lack of data on the toxicity of *α*-humulene highlights the need for further investigation.

β-caryophyllene is a bicyclic sesquiterpene widely distributed in the plant kingdom and has a pivotal role in the survival and evolution of higher plants. It has a woody, spicy aroma [42]. β-caryophyllene is approved by United States Food and Drug Administration and European agencies as food additive, taste enhancer and flavouring agent and termed as a phyto-cannabinoid [24,62]. Hashiesh et al. [63] reviewed the CB2R-selective pharmacological properties and therapeutic potential of β-caryophyllene such as cardioprotective [64], hepatoprotective [65], neuroprotective, nephroprotective, gastroprotective, chemo-preventive [66], antioxidant, anti-inflammatory, and immunomodulator. β-caryophyllene is a component of vegetables and an estimated daily intake of 10–200 mg of this lipophilic sesquiterpene could be a dietary factor that potentially modulates inflammatory and other pathophysiological processes via the endocannabinoid system. Consequently, the pharmacokinetics of β-caryophyllene in humans and its potential impact on health is yet to be explored [67].

β-Farnesene is the only naturally occurring isomer and constituent of various essential oils. It is an acyclic volatile sesquiterpene with a woody, sweet, citrus aroma and was first discovered in apple peels in the 1960s. It plays an important role as insect repellent and was the precursor to antimicrobial agents [42]. There is insufficient data available to truly evaluate the toxicity of β-farnesene. A single study demonstrated that this compound is neither cytotoxic nor genotoxic [38].

### 4.3. Uses of Hop Oils

Essential oils derived from *Humulus lupulus* (hop), have garnered attention for their diverse therapeutic potential, particularly due to the bioactive terpenes and terpenoids they contain. However, these oils present challenges due to their poor water solubility, low bioavailability, and high volatility, which can hinder their effectiveness when applied directly. Terpenes often require higher concentrations to achieve desired effects, as noted by Del Prado-Audelo et al. [57], who highlighted that these compounds exhibit reduced cell penetration and stability. To address these limitations, alternative delivery mechanisms such as micro- or nano-formulations have been proposed, as they improve the efficiency of terpene delivery while reducing the required dosage [68].

Despite their promise, the antimicrobial activity of hop oils can be inconsistent. For instance, studies have shown that hop essential oils exhibit minimal antimicrobial effects against Gram-negative bacteria such as *Escherichia coli* and *Enterococcus faecalis*, while showing more significant activity against Gram-positive bacteria such as *Bacillus subtilis* and *S. aureus* [69]. This limited potency, particularly against certain pathogens, underlines the need for further research into enhancing hop oil formulations. In addition, the hydrolates from hop oils, which are water-soluble fractions, demonstrate reduced antioxidant activity compared to the essential oils [6]. While hop oils are being explored as alternatives to antibiotics, studies such as those by Sabbatini et al. [15] have indicated their potential role in enhancing the effectiveness of antibiotics, rather than replacing them. This aligns with the growing interest in natural products as supplementary treatments for infections. However, as noted in reviews by Carbone & Gervasio [70] and Becker et al. [43], the lack of standardised extracts and robust clinical trials remains a significant challenge for the widespread therapeutic use of hop oils.

Hop oils and their constituents generally show low acute toxicity, with no significant adverse effects reported in either in vitro or in vivo studies [17]. Nevertheless, the complexity of hop-derived ingredients complicates the assessment of their toxicokinetics. While adverse effects are rare, careful quantification of individual components is essential for ensuring safe usage, especially in pharmaceutical applications. Duarte et al. [42] provided a comprehensive summary of the diverse uses of hop oils in pharmaceuticals, ranging from sleep aids to anti-inflammatory treatments, though the majority of related patents are filed in China, none in South Africa. These applications highlight the growing interest in hop oils’ therapeutic potential, but underscore the need for further studies to better understand their efficacy and safety profiles.

## 5. Conclusions

Essential oils were successfully distilled from fresh hop plant material (leaves, cones and bract), considered a waste product, with yields closely resembling hop oil content supplied in product information of pelletised hops available in the South African market. This work profiled the chemical composition of oils distilled from South African varieties African Queen, experimental XJA2/436, Southern Sublime, Southern Aroma, Southern Dawn, Southern Passion, Southern Promise, and Southern Star and confirmed them to be terpene rich, containing high fractions of myrcene, α-humulene, β-caryophyllene and β-farnesene. The noted diversity of chemical profiles and variability in fractions of minor other terpenic compounds among different varieties is promising for their application as aroma and mouthfeel enhancers in beer. Known bioactive properties of the major constituents make these oils a resource for application in fields such as nutraceutical and pharmaceutical research. Future research will include assessing antimicrobial- and toxicity properties, as well as determining the presence of pesticides and heavy metals in the extracted oils. The potential for use as terpene rich whole oils without the complicated legislation that regulates other essential oils such as cannabis oil, is promising for varieties that are high in β-caryophyllene namely Southern Aroma and Passion.

## Figures and Tables

**Table 1:** Functional group analysis and refractive index of essential oils distilled from 8 South African hop varieties.

Functional Group	Fraction (%) in Hop Varieties							
	Queen	XJA2/436	Sublime	Aroma	Dawn	Passion	Promise	Star
Alcohols	1.29	1.65	0.95	1.89	1.16	1.43	1.40	1.31
Aldehydes	0.04	0.11	0.06	0.21	0.14	0.21	0.48	0.26
Ethers	0.61	1.41	0.53	2.16	1.01	1.68	3.13	1.58
Esters	4.74	3.36	5.64	2.94	3.39	2.81	3.56	3.61
Ketones	2.25	0.91	2.24	3.52	3.51	2.36	2.90	2.41
Terpenes	86.93	89.81	85.94	83.97	85.88	84.80	82.20	85.81
Refractive index *	1.481	1.482	1.480	1.486	1.485	1.485	1.486	1.483

* Refractive index standard deviation = 0.0024.

**Table 2:** Chemical structures (skeleton), identifiers and toxicity characteristics of selected aliphatic and terpene compounds detected in hop oils from 8 South African varieties.

Compound, IUPAC Name, (Class), & Chemical Structure	Compound Summary	Toxicology	Fractions (%) in Hop Oils from This Study
**Methyl (4Z) decenoate** Synonym: Methyl E-4-decenoate IUPAC: Methyl (Z)-4-decenoate (Aliphatic ester) 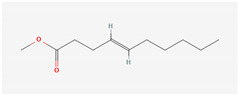	**CAS-No:** 93979-14-7 **Formula:** C_11_H_20_O_2_ **Molecular Weight:** 184.27 g/mol **PubChem ID:** *CID 5366840*	No acute oral, dermal or inhalation toxicity, or skin or eye irritation, or skin sensitisation were noted. No treatment-related effects were noted up to and including the limit dose of 1000 mg/kg bw/day after repeated oral exposure [27]. (in vivo) Estimated MSDI of 0.0012 µg/person/day with a threshold concern at 1800 µg/person/day [28].	African Queen: 0.74% Southern Star: 0.72% Southern Sublime: 0.69% Southern Dawn: 0.65% Southern Passion: 0.63% Southern Aroma: 0.61%% Southern Promise: 0.59% XJA2/436: 0.42%
**2-Methylbutyl isobutyrate** IUPAC: 2-methylbutyl 2-methylpropanoate (Aliphatic ester) ** 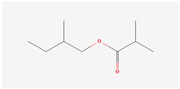 **	**CAS-No:** 2445-69-4 **Formula:** C_9_H_18_O_2_ **Molecular Weight:** 158.24 g/mol **PubChem ID:** *CID 97883*	When given to mice at a dose of 0.80 g/kg, narcosis is produced for about 8 h [29]. (in vivo) Estimated MSDI of 1.4 µg/person/day with a threshold concern at 1800 µg/person/day [28].	Southern Sublime: 1.35% African Queen: 0.98% XJA2/436: 0.77% Southern Star: 0.56% Southern Dawn: 0.52% Southern Promise: 0.43% Southern Aroma: 0.30% Southern Passion: 0.20%
**2-Undecanone** IUPAC: undecan-2-one (Aliphatic ketone) ** 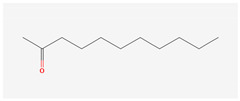 **	**CAS-No:** 112-12-9 **Formula:** C_11_H_22_O **Molecular Weight:** 170.29 g/mol **PubChem ID:** *CID 8163*	* Insufficient toxicity data: analogue = 2-heptanone (CAS # 110- 43-0) Genotoxicity: not expected (in vitro and in vivo) Repeated Dose Toxicity: NOAEL = 1087 mg/kg/day (in vivo) Skin Sensitisation: not a concern (in vivo) Local Respiratory Toxicity: No NOAEC available. Exposure is below the TTC (0.47 mg/day) Reference: [30]	Southern Dawn: 1.21% Southern Promise: 1.11% Southern Aroma: 0.99% Southern Star: 0.94% Southern Sublime: 0.88% Southern Passion: 0.87% African Queen: 0.75% XJA2/436: 0.43%
**2-Tridecanone** IUPAC: tridecan-2-one (Aliphatic ketone) ** 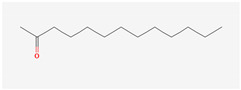 **	**CAS-No:** 593-08-8 **Formula:** C_13_H_26_O **Molecular Weight:** 198.34 g/mol **PubChem ID:** *CID 11622*	* Insufficient toxicity data: analogue = 2-heptanone (CAS # 110- 43-0) Genotoxicity: not expected (in vitro) Repeated dose toxicity: NOEL = 20 mg/kg/day (in vivo) Skin sensitisation: not a concern (in vivo, human) Respiratory toxicity: No NOAEC available. Exposure is below TTC (0.47 mg/day) Reference: [31]	African Queen: 0.77% Southern Dawn: 0.76% Southern Aroma: 0.75% Southern Sublime: 0.62% Southern Passion: 0.44% Southern Promise: 0.40% Southern Star: 0.39% XJA2/436: 0%
**Myrcene** IUPAC: 7-methyl-3-methylideneocta-1,6-diene (Monoterpene) 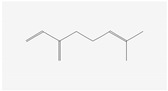	**CAS-No:** 123-35-3 **Formula:** C_10_H_16_ **Molecular Weight:** 136.23 g/mol **PubChem ID:** *CID 31253*	Genotoxicity: Not expected (in vivo) Repeated Dose Toxicity: NOAEL = 25 mg/kg/day (in vivo) Skin Sensitisation: None (in vivo) Local Respiratory Toxicity: No NOAEC available. Exposure is below the TTC. (1.4 mg/day) (in vivo) References: [32,33]	XJA2/436: 48.15% Southern Sublime: 42.15% African Queen: 40.57% Southern Star: 37% Southern Promise: 33.35% Southern Dawn: 31.94% Southern Passion: 30.65% Southern Aroma: 27.80%
**α-Humulene** IUPAC: (1Z,4Z,8E)-2,6,6,9-tetramethylcycloundeca-1,4,8-triene (Sesquiterpene) ** 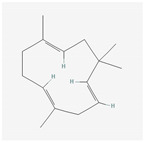 **	**CAS-No:** 6753-98-6 **Formula:** C_15_H_24_ **Molecular Weight:** 204.35 g/mol **PubChem ID:** *CID 5281520*	No RIFM toxicity review available. A single study by LeVoie et al. [34] noting marginal toxicity in rats exposed to clove smoke, highlighted in a recent review by Mendes de Lacerda Leite et al. [35].	Southern Dawn: 24.89% Southern Aroma: 24.07% Southern Passion: 22.75% Southern Promise: 22.54% Southern Star: 22.21% African Queen: 22.05% Southern Sublime: 19.96% XJA2/436: 19.52%
**β-Caryophyllene ** IUPAC: (1R,4E,9S)-4,11,11-trimethyl-8-methylidenebicyclo [7.2.0] undec-4-ene (Sesquiterpene) 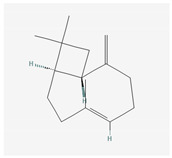	**CAS-No:** 87-44-5 **Formula:** C_15_H_24_ **Molecular Weight:** 204.35 g/mol **PubChem ID:** *CID 5281515*	Genotoxicity: None (in vivo and in vitro) Repeated Dose Toxicity: NOAEL = 1033 mg/kg/day (in vivo) Skin Sensitisation: None (in vivo also human) Local Respiratory Toxicity: No NOAEC available. Exposure is below the TTC (1.4 mg/day) (in vivo, human) Reference: [36]	Southern Passion: 13.73% Southern Aroma: 13.36% Southern Dawn: 12.15% Southern Promise: 11.38% Southern Star: 10.87% Southern Sublime: 9.94% African Queen: 8.93% XJA2/436: 8.47%
**β-Farnesene** (E)-7,11-dimethyl-3-methylenedodeca-1,6,10-triene (Sesquiterpene) 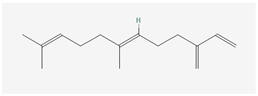	**CAS-No:** 18794-84-8 **Formula:** C_15_H_24_ **Molecular Weight:** 204.35 g/mol **PubChem ID:** *CID 5281517*	Genotoxicity: Not expected (in vitro) Repeated Dose Toxicity: No NOAEL available. Exposure is below the TTC (0.03 mg/g/day) (insufficient data available) Skin Sensitisation: NESIL = 3.7 mg/cm^2^ (in vivo and human studies) Local Respiratory Toxicity: No NOAEC available. Insufficient inhalation data available. Exposure is below the TTC (1.4 mg/day). References: [37,38]	African Queen: 7.57% Southern Star: 6% XJA2/436: 5.11% Southern Dawn: 4.82% Southern Promise: 4.34% Southern Aroma: 4.23% Southern Sublime: 2.08% Southern Passion: 1.84%

* In silico evaluation was conducted to determine read-across analogues with sufficient data for toxicological evaluation. Based on structural similarity, reactivity, metabolism, physical–chemical properties, and expert judgment (Research Institute for Fragrance Materials -RIFM international expert panel) and using the ECHA read-across assessment framework (RAAF) (ECHA, 2017). MSDI = Maximum Daily Intake; NESIL = No Expected Sensitisation Induction Level; NOEL = No Observed Effect Level; NOAEL = No Observed Adverse Effect Level; NOAEC = No Observed Adverse Effect Concentration; TTC = Threshold of Toxicological Concern.

## Data Availability

Data are contained within the article.

## Supplementary Materials

The following supporting information can be downloaded at: https://www.mdpi.com/article/10.3390/life15020282/s1, Supplementary Table S1: Chemical components in essential oils distilled from 8 South African hop varieties determined by GC-FID and MS.
