# Supplementary material for: Comparison of Major Compounds in Essential Oils Steam Distilled from Fresh Plant Material of South African Hop Varieties
Source: Life (Basel). 2025 Feb 12;15(2):282. doi: 10.3390/life15020282 (PMC11857115; doi:10.3390/life15020282)
Supplement: Supplementary file 1 [file life-15-00282-s001.zip › life-3386130-supplementary.pdf]

Supplementary Table S1: Chemical components in essential oils distilled from 8 South African hop varieties determined by GC-FID

			Queen	XJA2/436	Sublime	Aroma	Dawn	Passion	Promise	Star
**IUPAC names**	**Identification**	**Class**	**%**	**%**	**%**	**%**	**%**	**%**	**%**	**%**
(E)-hex-2-en-1-ol	(2E)-Hexenol	Aliphatic alcohol	0,01				0,01		0,01	
(Z)-hex-1-en-1-ol	(3Z)-Hexenol								0,01	
undecan-2-ol	2-Undecanol		0,01	0,02	0,03					
decan-1-ol	Decanol			0,01		0,05	0,15	0,03	0,09	0,05
ethanol	Ethanol					0,11	0,01	0,02		0,02
octan-1-ol	Octanol		0,01	0,01		0,01	0,02	0,01	0,03	0,01
oct-1-en-3-ol	Octen-3-ol		0,02	0,01	0,02	0,01	0,01	0,01	0,01	0,01
decanal	Decanal	Aliphatic aldehyde		0,01						
heptanal	Heptanal				0,01	0,02	0,02	0,02	0,04	0,01
hexanal	Hexanal			0,02	0,01	0,00	0,02	0,00	0,02	0,01
3-methylbutanal	Isovaleral				0,01		0,02			
nonanal	Nonanal			0,03		0,16	0,02	0,18	0,39	0,23
2-methylbutanal	2-Methylbutyral						0,02		0,00	
hexan-1-ol	Hexanol								0,01	
2-methylbutan-1-ol	2-Methylbutanol	Aliphatic ester			0,01					
2-methylbutyl 2-methylbutanoate	2-Methylbutyl 2-methylbutyrate		0,16	0,09	0,25	0,09	0,15			
2-methylbutyl acetate	2-Methylbutyl acetate		0,02	0,01	0,03	0,01	0,02	0,01	0,01	0,02
2-methylbutyl heptanoate	2-Methylbutyl heptanoate					0,14	0,11	0,15	0,13	0,12
2-methylbutyl 2-methylpropanoate	2-Methylbutyl isobutyrate		0,98	0,77	1,35	0,30	0,52	0,20	0,43	0,56

pentyl 2-methylpropanoate	Amyl isobutyrate			0,01		0,01	0,02		0,07	0,03
pentyl 3-methylbutanoate	Amyl isovalerate		0,14		0,19	0,07	0,10	0,04	0,07	0,11
butyl 2-methylpropanoate	Butyl isobutyrate			0,03					0,05	
heptyl acetate	Heptyl acetate		0,01							
heptyl 2-methylpropanoate	Heptyl isobutyrate		0,01	0,03	0,04					
heptyl propanoate	Heptyl propionate		0,02		0,02	0,03	0,04	0,02	0,04	0,02
3-methylbutyl butanoate	Isoamyl butyrate									0,02
hexyl acetate	Hexyl acetate		0,02	0,01	0,02	0,01	0,01	0,02	0,01	0,01
hexyl 2-methylpropanoate	Hexyl isobutyrate		0,02	0,01	0,03					
hexyl propanoate	Hexyl propionate		0,02	0,01	0,03					
3-methylbutyl 2-methylbutanoate	Isoamyl 2-methylbutyrate		0,01							
3-methylbutyl 2-methylpropanoate	Isoamyl isobutyrate		0,29	0,17	0,32	0,14	0,12		0,15	0,23
3-methylbutyl 3-methylbutanoate	Isoamyl isovalerate			0,08						
2-methylpropyl 2-methylbutanoate	Isobutyl 2-methylbutyrate		0,02		0,02	0,01	0,02	0,02	0,01	0,03
2-methylpropyl propanoate	isobutyl propionate			0,01						
2-methylpropyl 2-methylpropanoate	Isobutyl isobutyrate		0,12	0,14	0,25	0,04	0,09	0,04	0,10	0,06
2-methylpropyl 3-methylbutanoate	Isobutyl isovalerate		0,1	0,04	0,08	0,17	0,04	0,26	0,50	0,21
methyl (Z)-non-3-enoate	Methyl (3Z)-nonenoate		0,06	0,05	0,08	0,04	0,04	0,02	0,03	0,05
Methyl (E)-4-decenoate	Methyl (4E)-decenoate		0,02	0,02	0,03	0,02	0,02	0,02	0,02	0,02
Methyl (Z)-4-decenoate	Methyl (4Z)-decenoate		0,74	0,42	0,69	0,61	0,65	0,63	0,59	0,72
methyl 4-methylhex-2-enoate	Methyl 4-methylhex-2-enoate		0,01		0,01	0,07	0,07	0,06	0,12	0,10
methyl 4-methylhexanoate	Methyl 4-methylhexanoate		0,02		0,04	0,03	0,03			
methyl 5-methylhexanoate	Methyl 5-methylhexanoate		0,03		0,05			0,02		0,03
methyl 6-methylheptanoate	Methyl 6-methylheptanoate		0,06	0,08	0,14	0,07	0,10	0,08	0,10	0,08
methyl 6-methyloctanoate	Methyl 6-methyloctanoate		0,01	0,01	0,03					
methyl 7-methyloctanoate	Methyl 7-methyloctanoate		0,01	0,01		0,02	0,01	0,01	0,03	0,01
methyl 8-methyldecanoate	Methyl 8-methyldecanoate		0,06	0,04	0,13					
methyl 8-methylnonanoate	Methyl 8-methylnonanoate		0,05	0,03	0,09	0,03	0,03	0,02	0,03	0,04
methyl 9-methyldecanoate	Methyl 9-methyldecanoate			0,01						

methyl (2E,4E)-deca-2,4-dienoate	Methyl decadienoate isomer I		0,13	0,05	0,15	0,13	0,12	0,12	0,14	0,14
Methyl decanoate	Methyl decanoate		0,11	0,03	0,16	0,11	0,11	0,15	0,10	0,10
methyl dodecanoate	Methyl dodecanoate isomer I				0,16	0,11	0,11	0,14	0,11	0,17
methyl dodec-11-enoate	Methyl dodecenoate isomer I		0,21	0,12						
Methyl heptanoate	Methyl heptanoate		0,02	0,06	0,13			0,10	0,13	0,13
Methyl hexanoate	Methyl hexanoate		0,01	0,01	0,02	0,03	0,02	0,01	0,02	0,04
Methyl nonanoate	Methyl nonanoate		0,07	0,06	0,19	0,06	0,08	0,05	0,07	0,06
Methyl octanoate	Methyl octanoate		0,13	0,08	0,18	0,09	0,09	0,10	0,11	0,11
octyl 2-methylpropanoate	Octyl isobutyrate		0,01		0,02	0,01	0,02	0,01	0,01	
Octyl propionate	Octyl propionate			0,02		0,04	0,03	0,05	0,03	0,02
3-methylbut-2-enyl 2-methylpropanoate	Prenyl isobutyrate		0,08	0,04	0,01	0,02	0,01			
(2E)-2-, methylbut-2-en-1-yl 2-methylpropanoate	Tiglyl isobutyrate		0,02	0,02	0,02					
Methyl undecanoate	Methyl undecanoate					0,03	0,02	0,03	0,02	0,01
octyl ethanoate	Octyl acetate					0,01				
(Z)-6-Pentadecen-2-one	(6Z)-Pentadecen-2-one	Aliphatic ketone	0,16	0,09	0,11	0,13	0,11	0,14	0,11	0,10
(7Z)-tetradec-7-en-2-one	(7Z)-Tetradecen-2-one		0,02	0,01		0,13	0,11	0,13	0,11	0,10
methyl undec-10-enoate	10-Methyl-2-undecanone		0,02	0,01	0,04	0,03	0,04	0,01	0,03	0,03
decan-2-one	2-Decanone		0,09	0,07	0,2	0,40	0,22	0,09	0,17	0,13
dodecan-2-one	2-Dodecanone		0,02		0,02	0,40	0,15	0,14	0,12	0,08
pentadecan-2-one	2-Pentadecanone			0,05		0,11	0,08	0,08	0,11	0,09
tetradecan-2-one	2-Tetradecanone		0,01							
tridecan-2-one	2-Tridecanone		0,77		0,62	0,75	0,76	0,44	0,40	0,39
undecan-2-one	2-Undecanone		0,75	0,43	0,88	0,99	1,21	0,87	1,11	0,94
(3E)-5-Methyl-3-hexen-2-one	5-Methyl-(3E)-hexen-2-one						0,03			
6-methylhept-5-en-2-one	6-Methyl-5-hepten-2-one		0,02	0,02	0,02	0,02	0,02	0,02	0,02	0,02
8-methylnonan-2-one	8-Methyl-2-nonanone		0,03	0,01	0,05	0,03	0,04	0,01	0,05	0,03
2-decanone, 9-methyl	9-Methyl-2-decanone		0,02	0,05	0,04	0,14	0,25	0,10	0,18	0,08

(Z)-5-undecen-2-one	Undec-(5Z)-en-2-one		0,27	0,13	0,19	0,27	0,37	0,21	0,37	0,33
(Z)-undec-7-en-2-one	Undec-(7Z)-en-2-one		0,07	0,04	0,07	0,07	0,10	0,07	0,08	0,07
1,3-dimethyltrisulfane	Dimethyl trisulfide	Aliphatic sulfide		0,01						
Not known	Methanethiol 4-methylvalerate	Aliphatic thioester	0,01	0,01	0,03	0,02	0,02		0,01	
methyl 2-methylbutanethioate	S-Methylthiomethyl 2-methylbutyrate		0,1		0,04					
S-methyl 3-methylbutanethioate	S-Methylthiomethyl isovalerate					0,02	0,02	0,03	0,02	
Octane	Octane	Alkanes			0,02	0,06	0,09	0,05	0,19	0,05
2,4-dimethylhepta-2,4-diene	(E)-2,4-Dimethyl-2,4-heptadiene							0,01	0,02	
(3E)-nona-1,3-diene	(E)-1,3-Nonadiene		0,03		0,06	0,02	0,03			
nona-1,3,5-triene	1,3,5-Nonatriene isomer				0,02					
decanal	Decanal				0,02					
2,6-dimethyl-2,6-octadiene	Dimethyloctadiene isomer I		0,02	0,01	0,05					
(3E)-penta-1,3-diene	(E)-1,3-Pentadiene			0,01						
(3E,5E)-undeca-1,3,5-triene	(3E,5E)-Undeca-1,3-5-triene					0,03	0,03	0,01	0,04	0,04
(3E,5Z)-undeca-1,3,5-triene	(3E,5Z)-Undeca-1,3,5-triene			0,03						
1,4-Dimethyl-4-vinylcyclohexene	4-Ethenyl-1,4-bis(4-methyl-3-penten-1-yl)-cyclohexene	Diterpene		0,01	0,01	0,02			0,02	
Not known	5-Ethenyl-1,5-bis(4-methyl-3-penten-1-yl)-cyclohexene		0,01	0,01	0,01					0,03
5-(6-methylhepta-1,5-dien-2-yl)-1-(4-methylpent-3-en-1-yl)cyclohex-1-ene	meta-Camphorene		0,06	0,10	0,06	0,09	0,11	0,10	0,13	0,14
4-(6-methylhepta-1,5-dien-2-yl)-1-(4-methylpent-3-en-1-yl)cyclohex-1-ene	para-Camphorene		0,04	0,05	0,04	0,04	0,05	0,04	0,07	0,07

(6E)-2,6-Dimethyl-2,6-octadiene	(6E)-2,6-Dimethyl-2,6-octadiene	Monoterpene				0,04	0,02	0,02	0,01	0,02
(3E)-3,7-dimethylocta-1,3,6-triene	(E)-β-Ocimene		0,17	0,18	0,76	0,40	0,27	0,13	0,12	0,20
(3Z)-3,7-dimethylocta-1,3,6-triene	(Z)-β-Ocimene		0,02	0,02	0,03	0,40	0,02	0,01	0,01	0,01
2,2-dimethyl-3-methylidenebicyclo[2.2.1]heptane	Camphene		0,01	0,01		0,01	0,00	0,01	0,01	0,01
1-methyl-4- prop-1-en-2-ylcyclohexene	Limonene		0,14	0,21	0,17	0,14	0,15	0,14	0,18	0,18
7-methyl-3-methylideneocta-1,6-diene	Myrcene		40,57	48,15	42,15	27,80	31,94	30,65	33,35	37,00
1-isopropyl-4-methylbenzene	para-Cymene		0,01	0,01		0,01	0,01			
4-methylidene-1-propan-2-ylbicyclo[3.1.0]hexane	Sabinene		0,02	0,02	0,02	0,02	0,02			
1-methyl-4-(propan-2-ylidene)cyclohex-1-ene	Terpinolene		0,02	0,01	0,02					
2-methyl-5-(propan-2-yl)cyclohexa-1,3-diene	α-Phellandrene		0,01		0,01			0,01	0,03	0,01
(1S,5S)-2,6,6-Trimethylbicyclo[3.1.1]hept-2-ene ((−)-α-Pinene)	α-Pinene		0,08	0,08	0,06	0,05	0,06	0,06	0,08	0,08
1-methyl-4-(propan-2-yl)cyclohexa-1,3-diene	α-Terpinene		0,01		0,02	0,01	0,01	0,01		0,01
1-Isopropyl-4-methylbicyclo[3.1.0]hex-3-ene	α-Thujene			0,01			0,01	0,00	0,02	0,01
3-methylidene-6-propan-2-ylcyclohexene	β-Phellandrene		0,11	0,21	0,15					0,03
6,6-dimethyl-2-methylidenebicyclo[3.1.1]heptane	β-Pinene		0,54	0,70	0,58	0,45	0,55	0,48	0,60	0,56
1-methyl-4-(propan-2-yl)cyclohexa-1,4-diene	γ-Terpinene				0,03	0,01	0,02	0,02	0,01	0,02

(1R,2S,4R)-1,7,7-trimethylbicyclo[2.2.1]heptan-2-ol	Borneol	Monoterpenic alcohol	0,01		0,02					
2-[(2R,5S)-5-ethenyl-5-methyloxolan-2-yl]propan-2-ol	cis-Linalool oxide (fur,)		0,01							
(2E)-3,7-dimethylocta-2,6-dien-1-ol	Geraniol		0,3	0,28	0,13	0,07	0,05	0,10	0,05	0,23
(4S)-2-Methyl-6-methylideneocta-2,7-dien-4-ol	Ipsdienol		0,02	0,01	0,02					
3,7-dimethylocta-1,6-dien-3-ol	Linalool		0,17	0,16	0,13	0,10	0,13	0,10	0,18	0,13
(2Z)-3,7-dimethylocta-2,6-dien-1-ol.	Nerol		0,02	0,02	0,02	0,02	0,02			
4-methyl-1-(propan-2-yl)cyclohex-3-en-1-ol	Terpinen-4-ol		0,01							
p-Menth-1-en-8-ol 2-(4-Methylcyclohex-3-en-1-yl)propan-2-ol	α-Terpineol		0,02	0,01	0,04	0,01	0,02	0,01		
2-[(2S,5S)-5-ethenyl-5-methyloxolan-2-yl]propan-2-ol	trans-Linalool oxide (fur,)					0,01	0,01			
(4-propan-2-ylcyclohexa-1,4-dien-1-yl)methanol	1,4-para-Menthadien-7-ol			0,50						
(2E)-3,7-Dimethyl-2,6-octadienal	Geranial	Monoterpenic aldehyde	0,02	0,03	0,02	0,02	0,03	0,01	0,02	0,01
(2Z)-3,7-dimethylocta-2,6-dienal	Neral		0,02	0,02	0,01		0,01			
3,7-dimethylocta-2,6-dienyl 2-methylbutanoate	Geranyl 2-methylbutyrate	Monoterpenic ester	0,05	0,01	0,05					
[(2E)-3,7-dimethylocta-2,6-dienyl] acetate	Geranyl acetate		0,09	0,07	0,08	0,03	0,10	0,03	0,02	0,02
[(2E)-3,7-dimethylocta-2,6-dienyl] 2-methylpropanoate	Geranyl isobutyrate		0,19	0,07	0,24	0,14	0,21	0,17	0,14	0,15
[(2E)-3,7-dimethylocta-2,6-dienyl] 3-methylbutanoate	Geranyl isovalerate			0,02						

methyl (2E)-3,7-dimethylocta-2,6-dienoate	Methyl geranate		0,45	0,60	0,2	0,19	0,20	0,19	0,12	0,22
[(2Z)-3,7-dimethylocta-2,6-dienyl] 2-methylbutanoate	Neryl 2-methylbutyrate		0,01	0,01						
2,2-Dimethyl-3-(3-methylene-4-penten-1-yl)oxirane	6,7-Epoxymyrcene	Monoterpenic ether	0,02	0,04	0,02			0,01	0,01	
(2R)-2-ethenyl-2,6,6-trimethyloxane	cis-Dehydroxylinalool oxide		0,02	0,02		0,03	0,02			
methyl 2-phenylacetate	Methyl phenylacetate	Phenolic ester	0,04		0,03	0,01	0,01	0,01	0,02	0,00
(3E,6E)-3,7,11-Trimethyl-1,3,6,10-dodecatetraene	(3E,6E)-α-Farnesene	Sesquiterpene	0,38	0,38	0,32	0,26	0,21	0,29	0,15	0,24
(3Z,6E)-3,7,11-trimethyldodeca-1,3,6,10-tetraene	(3Z,6E)-α-Farnesene			0,45						
(6E)-7,11-dimethyl-3-methylidenedodeca-1,6,10-triene	(E)-β-Farnesene		7,57	5,11	2,08	4,23	4,82	1,84	4,34	6,00
(4*S*,4*aR*)-4,4*a*-dimethyl-6-prop-1-en-2-yl-2,3,4,5,6,7-hexahydro-1*H*-naphthalene	4,5-diepi-Aristolochene		0,02		0,06	0,09	0,06	0,11	0,04	0,03
5,8a-dimethyl-3-prop-1-en-2-yl-2,3,4,4a,7,8-hexahydro-1H-naphthalene	7-epi-α-Selinene			0,18						
(1S,4E,9S)-4,11,11-trimethyl-8-methylidenebicyclo[7.2.0]undec-4-ene	9-epi-Isocaryophyllene		0,03	0,02	0,03					
1,1,7-trimethyl-4-methylidene-2,3,4a,5,6,7,7a,7b-octahydro-1aH-cyclopropa[e]azulene	Aromadendrene		0,01	0,02	0,09					
(1S,9R)-10,10-dimethyl-2,6-dimethylidenebicyclo[7.2.0]undecan-5-ol	Caryophylla-4(12),8(13)-diene		0,38	0,05	0,42	0,62	0,52	0,12	0,13	0,11

(2*S*,4*aS*)-2,5-dimethyl-8-propan-2-yl-1,2,3,4,4*a*,7-hexahydronaphthalene	cis-Cadina-1(6),4-diene			0,03						
1,6-dimethyl-4-propan-2-yl-1,2,3,7,8,8a-hexahydronaphthalene	Epizonarene		0,09			0,20	0,15	0,29	0,12	0,13
(1E,5E)-1,5-dimethyl-8-propan-2-ylidenecyclodeca-1,5-diene	Germacrene B		0,17	0,10	0,87	0,48	0,90	0,40	0,49	0,44
(1E,6E,8S)-1-methyl-5-methylidene-8-propan-2-ylcyclodeca-1,6-diene	Germacrene D			0,06						
(1R,4Z,9S)-4,11,11-trimethyl-8-methylidenebicyclo[7.2.0]undec-4-ene	Isocaryophyllene		0,1	0,03	0,04	0,06	0,05	0,06	0,06	0,05
(3S,8aS)-5,8a-dimethyl-3-prop-1-en-2-yl-2,3,4,4a,7,8-hexahydronaphthalen-1-one	Selina-3,7(11)-diene		0,02	0,04	0,4	0,21	0,47	0,15	0,23	0,12
2-(2,3-dimethylbut-3-enyl)-3-ethyl-1,3-dimethylcyclohexene	Selina-4,11-diene		0,08	0,06	0,21	0,26	0,23	0,18	0,12	
(1R,4S)-4,7-dimethyl-1-propan-2-yl-1,2,3,4,5,6-hexahydronaphthalene	trans-Cadina-1(6),4-diene		0,02	0,01	0,03					
(1S,4R)-1,6-dimethyl-4-propan-2-yl-1,2,3,4-tetrahydronaphthalene	trans-Calamenene			0,30				0,08	0,22	0,10
(1S,5S,6R)-2,6-dimethyl-6-(4-methylpent-3-en-1-yl)bicyclo[3.1.1]hept-2-ene	trans-α-Bergamotene		0,34	0,25	0,1	0,25	0,28	0,11	0,32	0,31
(1S,5S,6R)-6-methyl-2-methylidene-6-(4-methylpent-3-enyl)bicyclo[3.1.1]heptane	trans-β-Bergamotene			0,02		0,16	0,12	0,20	0,08	0,07
(3R,4aS,5R)-4a,5-dimethyl-3-prop-1-en-2-yl-2,3,4,5,6,7-hexahydro-1H-naphthalene	Valencene		0,06	0,23	0,1	0,16	0,12	0,21	0,11	0,10

(1S)-1,6-dimethyl-4-propan-2-yl-1,2,3,7,8,8a-hexahydronaphthalene	Zonarene					0,30	0,48	0,28	0,15	0,19
(1S,4aR,8aR)-4,7-dimethyl-1-propan-2-yl-1,2,4a,5,6,8a-hexahydronaphthalene	α-Cadinene		0,17	0,16	0,24	0,28	0,30	0,28	0,23	0,22
(1S)-4,7-dimethyl-1-propan-2-yl-1,2-dihydronaphthalene	α-Calacorene		0,05		0,05					
1,3-dimethyl-8-propan-2-yltricyclo[4.4.0.02,7]dec-3-ene	α-Copaene		0,27	0,31	0,29	0,44	0,35	0,46	0,39	0,33
4,10-dimethyl-7-propan-2-yltricyclo[4.4.0.01,5]dec-3-ene	α-Cubebene		0,07	0,08	0,05					
(1Z,4Z,8E)-2,6,6,9-tetramethylcycloundeca-1,4,8-triene	α-Humulene		22,05	19,52	19,96	24,07	24,89	22,75	22,54	22,21
(1S,4aS,8aR)-4,7-dimethyl-1-propan-2-yl-1,2,4a,5,6,8a-hexahydronaphthalene	α-Muurolene		0,29	0,76	0,34	0,45	0,36	0,44	0,39	0,34
(3R,4aR,8aR)-5,8a-dimethyl-3-prop-1-en-2-yl-2,3,4,4a,7,8-hexahydro-1H-naphthalene	α-Selinene		0,52	0,37	1,75	2,34	1,61	3,34	1,22	1,09
(1S,6R,7R,8S)-1,3-dimethyl-8-propan-2-yltricyclo[4.4.0.02,7]dec-3-ene	α-Ylangene		0,09	0,08	0,1	0,13	0,11	0,14	0,12	0,10
(4S)-1-methyl-4-(6-methylhepta-1,5-dien-2-yl)cyclohexene	β-Bisabolene		0,11	0,07	0,04	0,07	0,07	0,04	0,06	0,08
(1S,2R,6S,7R,8S)-1-methyl-5-methylidene-8-propan-2-yltricyclo[5.3.0.02,6]decane	β-Bourbonene		0,09	0,14	0,11	0,39	0,18	0,47	0,27	0,17
7-methyl-4-methylidene-1-propan-2-yl-2,3-dihydro-1H-naphthalene	β-Calacorene		0,04		0,01	0,03		0,04	0,02	0,02

(1R,4E,9S)-4,11,11-trimethyl-8-methylidenebicyclo[7.2.0]undec-4-ene	β-Caryophyllene		8,93	8,47	9,94	13,36	12,15	13,73	11,38	10,87
(1R,2S,6S,7S,8S)-1-methyl-3-methylidene-8-propan-2-yltricyclo[4.4.0.02,7]decane	β-Copaene			0,32				0,52	0,41	0,36
(1S,2S,4R)-1-ethenyl-1-methyl-2,4-bis(prop-1-en-2-yl)cyclohexane	β-Elemene		0,04	0,04	0,1	0,19	0,10	0,24	0,14	0,11
(3S,4aR,8aS)-8a-methyl-5-methylidene-3-prop-1-en-2-yl-1,2,3,4,4a,6,7,8-octahydronaphthalene	β-Selinene		0,4	0,27	1,36	1,88	1,24	2,74	1,00	0,81
1-methyl-3-methylidene-8-propan-2-yltricyclo[4.4.0.02,7]decane	β-Ylangene		0,13	0,16	0,16	0,02		0,02	0,18	0,01
7-methyl-4-methylidene-1-propan-2-yl-2,3,4a,5,6,8a-hexahydro-1H-naphthalene	γ-Cadinene		0,97	0,96	0,97	1,42	1,06	1,44	1,13	1,08
(1S)-4,7-dimethyl-1-propan-2-yl-1,2,3,5,6,8a-hexahydronaphthalene	δ-Amorphene		0,1		0,08	0,11	0,10	0,14	0,03	0,07
(1S,8aR)-4,7-dimethyl-1-propan-2-yl-1,2,3,5,6,8a-hexahydronaphthalene	δ-Cadinene		1,52	0,98	1,52	1,93	1,66	1,96	1,41	1,59
(1S,2R,3R,6R,8R)-6-methyl-7-methylidene-3-propan-2-yltricyclo[4.4.0.02,8]decane	Sativene					0,06		0,04	0,06	0,08
(8S)-3,8-dimethyl-5-propan-2-yl-1,2,3,7,8,8a-hexahydronaphthalene	trans-Muurola-3					0,02	0,03	0,02	0,01	0,02
(2E,6E)-3,7,11-trimethyldodeca-2,6,10-trien-1-ol	(2E,6E)-Farnesol	Sesquiterpenic alcohol		0,03	0,01	0,01		0,02	0,02	
(2E,6Z)-3,7,11-trimethyldodeca-2,6,10-trien-1-ol	(2E,6Z)-Farnesol		0,07							

(3Z,5R)-4,11,11-trimethyl-8-methyl idenebicyclo[7.2.0]undec-3-en-5-ol	(3Z)-Caryophylla-3,8(13)-dien-5β-ol		0,02		0,01	0,07	0,01	0,08	0,03	0,03
(6E)-3,7,11-trimethyldodeca-1,6,10-trien-3-ol	(E)-Nerolidol		0,05	0,05		0,08	0,08	0,08	0,08	0,02
(1R,4R,4aR,8aR)-4,7-dimethyl-1-propan-2-yl-2,3,4,5,6,8a-hexahydro-1H-naphthalen-4a-ol	1,10-diepi-Cubenol			0,05			0,08	0,05		
(1S,3R,4S,6R,7R)-3,7-Dimethyl-1-(propan-2-yl)bicyclo[4.4.0]dec-10-en-4-ol	10-epi-Cubenol		0,03		0,03					
(1R,4S,4aR,8aS)-1-Isopropyl-4,7-dimethyl-1,3,4,5,6,8a-hexahydro-4a(2H)-naphthalenol	1-epi-Cubenol		0,04		0,05					
1S,5R,9R)-10,10-dimethyl-2,6-dimethylidenebicyclo[7.2.0]undecan-5-ol	Caryophylladienol II		0,02	0,04	0,01	0,08	0,02	0,07	0,04	0,04
(5S,6R,9S,10R)-3,6-dimethyl-9-propan-2-ylspiro[4.5]dec-3-en-10-ol	Gleenol		0,02	0,01	0,01	0,03	0,01	0,03	0,02	0,01
2-[(3S,5R,6R)-6,10-dimethylspiro[4.5]dec-9-en-3-yl]propan-2-ol	Hinesol		0,02	0,02	0,02					
(4Z,8Z)-6,6,9-trimethyl-2-methylidenecycloundeca-4,8-dien-1-ol	Humulenol II		0,04	0,08						
(3E,7E)-1,5,5,8-tetramethylcycloundeca-3,7-dien-1-ol.	Humulol		0,07	0,03	0,02	0,03	0,02	0,02	0,03	0,02

(1S,2S,4aR,8aS)-4a-methyl-8-methylidene-2-propan-2-yl-1,2,3,4,5,6,7,8a-octahydronaphthalen-1-ol	Junenol		0,02	0,06	0,02	0,27	0,00	0,06	0,08	0,07
1,4a-dimethyl-7-propan-2-ylidene-3,4,5,6,8,8a-hexahydro-2H-naphthalen-1-ol	Juniper camphor		0,01	0,03	0,02	0,03	0,02	0,01	0,03	0,01
(1S,4aR,7R,8aR)-1,4a-dimethyl-7-prop-1-en-2-yl-2,3,4,5,6,7,8,8a-octahydronaphthalen-1-ol	Neointermedeol				0,04	0,11	0,07	0,09	0,08	0,06
1,6-dimethyl-4-propan-2-yl-3,4,4a,7,8,8a-hexahydro-2H-naphthalen-1-ol	α-Cadinol		0,11	0,05	0,05	0,10	0,05	0,08	0,07	0,09
2-[(2R,4aR,8aR)-4a,8-dimethyl-2,3,4,5,6,8a-hexahydro-1H-naphthalen-2-yl]propan-2-ol	α-Eudesmol				0,04	0,04	0,04	0,03	0,04	
(1R,4R,4aR,8aS)-1,6-dimethyl-4-propan-2-yl-3,4,4a,7,8,8a-hexahydro-2H-naphthalen-1-ol	α-Muurolol		0,03	0,04	0,02					
2-[(2R,4aR,8aS)-4a-methyl-8-methylidene-1,2,3,4,5,6,7,8a-octahydronaphthalen-2-yl]propan-2-ol	β-Eudesmol				0,03	0,06	0,05	0,05	0,03	0,05
2-[(2R,4aR)-4a,8-dimethyl-2,3,4,5,6,7-hexahydro-1H-naphthalen-2-yl]propan-2-ol	γ-Eudesmol		0,01		0,05	0,11	0,06	0,11	0,10	0,08
(1R,4S,4aS,8aS)-1,6-dimethyl-4-propan-2-yl-3,4,4a,7,8,8a-hexahydro-2H-naphthalen-1-ol	τ-Cadinol		0,1	0,09	0,08	0,20	0,08	0,15	0,12	0,14

(1S,4S,4aR,8aS)-1,6-dimethyl-4-propan-2-yl-3,4,4a,7,8,8a-hexahydro-2H-naphthalen-1-ol	τ-Muurolol		0,02	0,03	0,03	0,04	0,03	0,03	0,04	0,04
(1R,4R)-1,6-dimethyl-4-propan-2-yl-3,4-dihydro-2H-naphthalen-1-ol	cis-Calamenen-10-ol					0,06	0,02	0,07	0,05	0,01
(2R,4aS,7R)-1,4a-dimethyl-7-prop-1-en-2-yl-3,4,5,6,7,8-hexahydro-2H-naphthalen-2-ol	Cyperol					0,12	0,09	0,09	0,14	0,11
6-methyl-2-(4-methylcyclohex-3-en-1-yl) hept-5-en-2-ol	α-Bisabolol									0,07
4,4,8-trimethyltricyclo[6.3.1.02,5] dodecane-1,9-diol	Caryolane-1-9β-diol					0,02		0,01	0,01	0,02
(3Z)-4,8,11,11-tetramethylbicyclo[7.2.0] undec-3-en-5-ol	Caryophyllenyl alcohol			0,01						
(1R,4R,6R,10S)-4,12,12-trimethyl-9-methylidene-5-oxatricyclo[8.2.0.$0^{4,6}$]dodecane	Caryophyllene oxide	Sesquiterpenic ether	0,21	0,48	0,19	0,92	0,37	0,80	1,18	0,64
(1R,4S,6S,10R)-4,12,12-trimethyl-9-methylene-5-oxatricyclo[8.2.0.$0^{4,6}$]dodecane	Caryophyllene oxide isomer		0,02	0,06	0,02	0,09	0,06	0,06	0,20	0,07
(4E,7E)-1,5,9,9-tetramethyl-12-oxabicyclo[9.1.0]dodeca-4,7-diene	Humulene 9,10-epoxide		0,01	0,06		0,11	0,05	0,09	0,06	0,07
(3Z,7E)-1,5,5,8-tetramethyl-12-oxabicyclo[9.1.0]dodeca-3,7-diene	Humulene epoxide I		0,05	0,08	0,04	0,12	0,05	0,08	0,13	0,09
(1R,3E,7E,11R)-1,5,5,8-tetramethyl-12-oxabicyclo[9.1.0]dodeca-3,7-diene	Humulene epoxide II		0,26	0,63	0,24	0,80	0,41	0,54	1,45	0,66

(1S,10R)-4,12,12-trimethyl-9-methylidene-5-oxatricyclo[8.2.0.04,6]dodecane	Isocaryophyllene epoxide B		0,02	0,04	0,02	0,09	0,04	0,09	0,10	0,04
6,10,14-trimethylpentadecan-2-one	Phytone	Terpenic ketone				0,05	0,01	0,06	0,03	0,02
2-Ethylfuran	2-Ethylfuran	Furan					0,01			
